# Time-varying metoprolol exposure and the risk of acute kidney injury: a multicenter retrospective cohort study

**DOI:** 10.3389/fphar.2026.1899992

**Published:** 2026-10-22

**Authors:** Zhengfu Li, Hongkai Zha, Qingyun Gong, Meixian Wu, Yanyun She, Fan Xu

**Affiliations:** 1 920th Hospital of the Joint Logistics Support Force of the Chinese People’s Liberation Army, Kunming, Yunnan, China; 2 Department of Pharmacy, Guandu District People’s Hospital, Kunming, Yunnan, China

**Keywords:** acute kidney injury, drug safety, metoprolol, pharmacoepidemiology, time-varying exposure

## Abstract

**Background:**

Evidence regarding the association between metoprolol use and acute kidney injury (AKI) remains limited, particularly because of time-varying exposure, cardiovascular confounding, and uncertain temporal ordering. We evaluated the association between recent metoprolol exposure and incident AKI using a longitudinal time-varying framework.

**Methods:**

This multicenter retrospective longitudinal cohort study included adult inpatients from three Chinese hospitals between January 2020 and December 2024. Eligible patients had an estimated glomerular filtration rate ≥60 mL min^-1^·1.73 m^-2^ at cohort entry and sufficient serial serum creatinine (Scr) and medication execution records. Metoprolol was modeled as a lag-1 time-varying exposure, relating use on hospital day *t* to AKI risk on day *t*+1. Incident AKI was identified using rolling KDIGO Scr criteria. Time-varying Cox regression adjusted for demographic characteristics, baseline renal function, major comorbidities, baseline severity-related factors, and lagged concomitant medication exposures. Sensitivity and complementary analyses evaluated the robustness of the findings.

**Results:**

Among 291,049 screened hospitalizations, 20,333 patients contributed 284,470 risk intervals, and 1,050 developed incident AKI. Lag-1 metoprolol exposure was associated with a higher AKI hazard in the unadjusted model (HR, 2.53; 95% CI, 2.13–3.00). The association was substantially attenuated after adjustment but remained statistically significant in the primary model (HR, 1.37; 95% CI, 1.05–1.79; P = 0.022). Prespecified follow-up-time analyses suggested that the association was concentrated during the first 72 h after cohort entry. Sensitivity analyses generally yielded estimates in the same direction, although the magnitude and statistical significance varied across analytical specifications; the association was attenuated to non-significance in the 2-day lag analysis (HR, 1.27; 95% CI, 0.96–1.68) and after additional adjustment for vasopressor use (HR, 1.29; 95% CI, 0.98–1.70). No consistent monotonic dose–response relationship was observed.

**Conclusion:**

Recent metoprolol exposure was associated with a modestly higher hazard of incident AKI in the primary adjusted analysis. However, attenuation after adjustment, temporal heterogeneity, sensitivity to exposure timing and confounding control, and the absence of a consistent monotonic dose–response pattern suggest that residual confounding and clinical treatment context may contribute to the observed association. These findings should be interpreted as a hypothesis-generating short-term AKI safety signal rather than evidence of direct metoprolol nephrotoxicity or causality.

## 1 Introduction

Acute kidney injury (AKI) is a common clinical syndrome characterized by a rapid decline in renal function over hours to days, resulting in the accumulation of nitrogenous waste products—such as creatinine and urea nitrogen—and disturbances in fluid, electrolyte, and acid-base balance (Liu et al., 2024). Medications are a well-recognized cause of AKI, with approximately 30% of cases attributed to preventable adverse drug events (Fernández-Llaneza et al., 2025). Numerous pharmacological agents are established risk factors for AKI, notably the concomitant use of nonsteroidal anti-inflammatory drugs (NSAIDs), renin-angiotensin system (RAS) inhibitors, and diuretics—commonly termed “triple therapy”—alongside specific chemotherapeutic and antimicrobial drugs (Perazella, 2019; Calvo et al., 2023).

Metoprolol is a selective β1-adrenergic receptor blocker widely used in the treatment of hypertension, angina pectoris, and arrhythmias, and is among the most commonly prescribed β-blockers globally (López-Sendón et al., 2004; Yan et al., 2024). Despite its extensive clinical use, evidence regarding a potential link to an elevated AKI risk remains inconclusive. No reports of AKI have been documented in its prescribing information. However, pharmacovigilance data have raised concerns. An analysis of the U.S. FDA Adverse Event Reporting System (FAERS) by Yan et al. (2024) identified a significant pharmacovigilance signal associating metoprolol with BRASH syndrome—a clinical complex characterized by bradycardia, renal failure, atrioventricular block, shock, and hyperkalemia—with a notably high reporting odds ratio (ROR = 391.285 based on 186 cases). Recent pharmacoepidemiological studies suggest this syndrome may be a potential adverse effect of β-blocker therapy, particularly in susceptible patients (Shah et al., 2022; Majeed et al., 2023).

In a prior large-sample study involving 34,068 patients, we applied the Apriori association rule algorithm to electronic medical record data to identify potential drug-AKI associations. The analysis confirmed that AKI was strongly associated with a wide range of medications that have been previously reported to be nephrotoxic. Notably, beyond these expected findings, metoprolol also demonstrated a strong association with AKI, which was an unexpected signal (Zha et al., 2026).

To further evaluate this signal while addressing the temporal and clinical complexities of inpatient metoprolol use, we conducted a multicenter retrospective longitudinal cohort study using a time-varying exposure framework. Metoprolol administration was reconstructed at the hospital-day level and modeled as a lag-1 time-varying exposure, relating treatment on hospital day *t* to the risk of incident AKI on day *t*+1. We further accounted for cardiovascular comorbidities, acute illness severity, concomitant medications, and other clinically relevant confounders, and performed sensitivity, temporal-heterogeneity, multicenter, dose-related, and subgroup analyses to assess the robustness and clinical context of the observed association.

## 2 Materials and methods

### 2.1 Study design and population

This multicenter retrospective longitudinal cohort study was conducted using electronic medical records from three hospitals in China: the 920th Hospital of the Joint Logistics Support Force of the People’s Liberation Army, Kunming Guandu District People’s Hospital, and Zhaotong First People’s Hospital. The source population comprised 291,049 patients hospitalized at the participating institutions between January 2020 and December 2024. Data were retrieved from the electronic medical record systems of the participating hospitals and included demographic characteristics, hospitalization-linked diagnosis records, laboratory measurements, vital signs, hospitalization and ICU records, and medication administration records.

Patients were eligible for inclusion if they were aged ≥18 years and had sufficient renal function measurements to establish baseline renal function and permit longitudinal ascertainment of incident AKI. Specifically, patients were required to have a valid baseline serum creatinine (Scr) measurement with a corresponding laboratory-reported estimated glomerular filtration rate (eGFR), temporally matched within 1 h, and at least one subsequent Scr measurement after cohort entry during hospitalization. To establish a cohort with preserved renal function at study entry, the corresponding baseline eGFR was required to be ≥ 60 mL min^-1^·1.73 m^-2^. Demographic information, including age and sex, and structured diagnostic records were required for cohort characterization and covariate ascertainment; patients with unknown ethnicity were retained and classified as an Unknown/Missing category. Patients were also required to have sufficiently complete medication execution records to reconstruct hospital-day–level exposure to metoprolol and relevant concomitant medications during follow-up.

Patients were excluded if they had documented pre-existing chronic kidney disease or other chronic renal conditions, including nephrotic syndrome, chronic renal insufficiency, or chronic renal failure, identified from structured diagnostic records using corresponding International Classification of Diseases, 10th Revision (ICD-10) codes. This criterion was used to define a cohort with preserved renal function at entry and was not intended to classify patients with a single eGFR value < 60 mL min^-1^·1.73 m^-2^ as having chronic kidney disease. Patients with insufficient clinical or laboratory information to determine cohort eligibility or ascertain the study outcome, or with incomplete medication administration records that precluded reliable reconstruction of medication exposure, were also excluded.

To better reflect real-world inpatient care and avoid excessive selection of the study population, concomitant use of potentially nephrotoxic medications was not used as an exclusion criterion. Patients receiving these medications were retained in the cohort, and their exposure was accounted for as a potential confounding factor in the subsequent longitudinal analyses. Cardiovascular diseases, acute illness characteristics, and other clinical factors potentially associated with both metoprolol treatment and AKI risk were likewise retained and addressed through covariate adjustment.

The study protocol was reviewed and approved by the ethics committees of the three participating hospitals (approval Nos. 2025-052(Ke)-01, 2025070301, and 202507220221). The 920th Hospital of the Joint Logistics Support Force of the Chinese People’s Liberation Army served as the coordinating institution for this multicenter study. The participating hospitals conducted the study in accordance with the approved multicenter protocol. The requirement for informed consent was waived by the ethics committees of the participating institutions because of the retrospective nature of the study and the use of de-identified clinical data. No participants were contacted for the purposes of this study. The study was conducted and reported in accordance with the Strengthening the Reporting of Observational Studies in Epidemiology (STROBE) guidelines.

### 2.2 Cohort entry and follow-up

Cohort entry was defined as the date and time of the first available serum creatinine (Scr) measurement after hospital admission. The corresponding Scr value was designated as the baseline Scr. Baseline eGFR was obtained directly from the electronic laboratory report, and the Scr and eGFR records were required to be temporally matched within 1 h to ensure that they represented the same baseline renal-function assessment. Laboratory-reported eGFR values were retained as originally recorded and were not recalculated, truncated, or capped in the primary analysis. Patients entered the primary analytic cohort only if the corresponding reported eGFR was ≥60 mL min^-1^·1.73 m^-2^ and no documented pre-existing chronic or structural kidney disease was identified. Eligibility for the incident AKI risk set was determined using the available renal function information and the prespecified AKI ascertainment criteria described below.

Follow-up commenced at cohort entry and continued until the earliest occurrence of incident AKI, hospital discharge, in-hospital death, or the end of the available observation period. All subsequent Scr measurements obtained during hospitalization were evaluated sequentially for ascertainment of the first incident AKI event. The original date and time of each Scr measurement were retained to preserve the temporal ordering of renal function changes during follow-up.

For the longitudinal analysis, follow-up time was organized into hospital-day intervals beginning at cohort entry. Each patient contributed person-time while remaining at risk for a first incident AKI event. Metoprolol exposure and time-varying covariates were updated according to their status during the corresponding hospital-day intervals using day-level medication execution records and time-stamped clinical measurements. For patients who did not develop AKI, follow-up was censored at hospital discharge, in-hospital death, or the end of available observation, whichever occurred first.

### 2.3 Metoprolol exposure

Metoprolol exposure was ascertained from electronic medication execution records. Because exact within-day administration timestamps were not consistently available across the participating hospitals, exposure was reconstructed at the hospital-day level. A hospital day was classified as exposed if at least one metoprolol administration was documented as executed on that day and as unexposed if no executed metoprolol administration was recorded. Medication orders or prescriptions without evidence of execution were not considered as exposure.

For the primary analysis, metoprolol exposure was modeled as a daily time-varying current-use variable with a 1-hospital-day lag. Exposure status on hospital day *t* was applied to the AKI risk interval on hospital day *t*+1. Accordingly, individual patients could contribute both unexposed and exposed person-time during hospitalization according to their day-level medication execution history.

The 1-hospital-day lag was prespecified to establish temporal separation between exposure ascertainment and subsequent AKI ascertainment and to reduce the potential for reverse temporal attribution when metoprolol treatment and AKI ascertainment occurred on the same hospital day. Metoprolol exposure recorded on or after the first ascertainment of AKI did not contribute to the exposure history for that event.

Alternative hospital-day–level exposure definitions were evaluated in sensitivity and exploratory analyses, including same-day exposure and a 2-hospital-day lag. Additional analyses of temporal heterogeneity were conducted using follow-up-time categories since cohort entry rather than exact administration-to-event elapsed-time intervals because within-day administration ordering could not be reliably reconstructed. Details of these analyses are provided in the Statistical analysis section.

### 2.4 AKI outcome ascertainment

The primary outcome was the first occurrence of incident acute kidney injury (AKI) during hospitalization. AKI was ascertained using serial serum creatinine (Scr) measurements according to the serum creatinine criteria of the Kidney Disease: Improving Global Outcomes (KDIGO) Clinical Practice Guideline (KDIGO Acute Kidney Injury Work Group, 2012). AKI was defined as an increase in Scr of ≥26.5 μmol/L (0.3 mg/dL) within 48 h or an increase to ≥1.5 times the reference Scr value within the preceding 7 days. Because urine output was not routinely and consistently recorded in the electronic medical records of the participating hospitals, the urine-output criterion was not used for AKI ascertainment (Guthrie et al., 2022).

All available Scr measurements obtained after cohort entry were ordered chronologically and evaluated sequentially. For each subsequent Scr measurement, all preceding valid Scr values within the prior 48 h and prior 7 days were evaluated using rolling time windows. For the 48-h criterion, AKI was identified when the current Scr value had increased by ≥ 26.5 μmol/L relative to an eligible preceding measurement within 48 h. For the 7-day criterion, AKI was identified when the current Scr value was ≥1.5 times an eligible preceding Scr value obtained within the previous 7 days. Scr measurements separated by more than 7 days were not directly compared for the purpose of satisfying the 7-day relative-increase criterion. The first Scr measurement used to define cohort entry and baseline renal function was not used as a fixed reference for all subsequent AKI assessments; reference Scr values were updated dynamically according to the applicable 48-h and 7-day windows.

The AKI event time was defined as the date and time of the first Scr measurement that satisfied either of the prespecified KDIGO serum creatinine criteria. Accordingly, this time represents the first ascertainment of AKI based on the available laboratory data rather than the exact biological onset of renal injury. Once a patient met the AKI criteria, follow-up for the primary time-to-first-event analysis was terminated, and subsequent Scr measurements were not used to define additional AKI events.

The original date and time of each Scr measurement were retained to establish the temporal sequence between renal function changes and metoprolol administration. For the primary daily time-varying analysis, the first incident AKI event was assigned to the hospital day on which the qualifying Scr measurement was obtained. Patients who did not meet either KDIGO serum creatinine criterion during follow-up were considered free of an ascertained AKI event until censoring.

### 2.5 Covariates and confounding factors

Potential confounding factors were prespecified based on their clinical relevance to both metoprolol treatment and the risk of AKI. Covariates included demographic characteristics, cardiovascular and other comorbidities, hemodynamic status, baseline renal function, indicators of acute illness severity, and concomitant medication exposure. Covariates were classified as baseline or time-varying according to whether their status was fixed at cohort entry or could change during follow-up.

Demographic characteristics included age, sex, and ethnicity. Major pre-existing comorbidities were ascertained from hospitalization-linked diagnosis records using prespecified International Classification of Diseases, 10th Revision (ICD-10) code groups supplemented by diagnosis-text matching. Hypertension was identified using ICD-10 codes I10–I13 and I15; heart failure using I50, I11.0, I13.0, and I13.2; coronary artery disease using I20–I25; myocardial infarction using I21, I22, and I25.2; and atrial fibrillation using I48. A comorbidity was considered present when a qualifying diagnostic code or corresponding diagnosis term was identified in the available hospitalization-linked diagnosis records. The available diagnosis files did not consistently distinguish admission, discharge, principal, or secondary diagnosis status. Comorbidity ascertainment was not supplemented using outpatient medical histories, longitudinal problem lists, medication proxies, or diagnoses from prior hospitalizations.

Vital signs included systolic blood pressure (SBP), diastolic blood pressure (DBP), mean arterial pressure (MAP), and heart rate. Measurements available at cohort entry were used to characterize initial hemodynamic status, whereas subsequent measurements were retained with their original timestamps for longitudinal assessment. Tachycardia was prespecified as a heart rate >100 beats/min, and elevated blood pressure was defined as SBP ≥140 mmHg and/or DBP ≥90 mmHg. Hypotension was additionally evaluated as an indicator of hemodynamic instability during follow-up. Hypertension as a pre-existing comorbidity was identified from hospitalization-linked diagnosis records and was defined separately from inpatient blood-pressure measurements; elevated blood pressure during hospitalization alone was not used to classify a patient as having hypertension.

Baseline renal function was characterized using the first available serum creatinine (Scr) measurement after hospital admission and the corresponding estimated glomerular filtration rate (eGFR) reported by the hospital laboratory. eGFR values were extracted directly from the electronic laboratory reports and were not recalculated for the purposes of this study. For cohort eligibility, an eGFR ≥60 mL min^-1^·1.73 m^-2^ was considered preserved renal function, whereas an eGFR <60 mL min^-1^·1.73 m^-2^ was considered reduced renal function (Levey et al., 2005; 2011).

Indicators of acute illness severity included sepsis, hypotension, vasopressor use, and intensive care unit (ICU) status. These variables were ascertained from condition-specific hospital data sources. Sepsis was ascertained from hospital-provided sepsis-related diagnosis records rather than re-identified from the complete diagnosis database using a single study-defined ICD-10 code range. The original diagnosis dates were retained and used to distinguish baseline from follow-up sepsis status. Hypotension was ascertained from hospital-provided hypotension records containing the date of measurement and a hypotension indicator. ICU status was reconstructed from ICU admission and discharge interval records, and vasopressor exposure was identified from valid executed medication orders according to the recorded administration dates. For longitudinal analyses, dated sepsis, hypotension, ICU, and vasopressor records were used to construct baseline, current-day, and lagged hospital-day-level variables as applicable. Baseline acute-severity variables were defined using information available during the prespecified baseline period, whereas corresponding hospital-course variables retained their original timing and were reconstructed at the hospital-day level for use as time-varying covariates in additional analyses where applicable.

Concomitant medication exposure was ascertained from medication administration records using hospital-day–level medication execution records. Prespecified medication covariates included renin–angiotensin system (RAS) inhibitors, defined as angiotensin-converting enzyme inhibitors (ACEIs), angiotensin receptor blockers (ARBs), and angiotensin receptor–neprilysin inhibitors (ARNIs), as well as diuretics, other β-blockers, potentially nephrotoxic medications, and contrast agents. For descriptive purposes, ACEI, ARB, and ARNI use were summarized separately, whereas these agents were combined into a single RAS inhibitor variable for multivariable modeling. Potentially nephrotoxic medications included vancomycin, gentamicin, tobramycin, amikacin, piperacillin/tazobactam, colistin, sulfamethoxazole/trimethoprim, lithium, acyclovir, tacrolimus, cyclosporine, etoricoxib, diclofenac, and ibuprofen. These medication exposures were retained as time-varying covariates rather than being used as exclusion criteria. The number of concomitant medications, defined as the number of distinct medication classes administered excluding metoprolol, was additionally used as an indicator of overall medication burden.

In the longitudinal analyses, baseline covariates remained fixed throughout follow-up, whereas time-varying clinical and medication covariates were updated according to the corresponding hospital-day intervals. To maintain temporal ordering, only covariate information available before the risk interval under evaluation was used for adjustment, and time-varying covariates were lagged as appropriate to correspond with the lagged metoprolol exposure definition. Information occurring after the first ascertainment of AKI was not used in models evaluating the risk of that event.

### 2.6 Statistical analysis

#### 2.6.1 Descriptive analysis

Continuous variables were summarized as means with standard deviations (SDs) or medians with interquartile ranges (IQRs), as appropriate according to their distributions. Categorical variables were summarized as frequencies and percentages. The distribution of continuous variables was assessed using graphical methods and the Shapiro–Wilk test where appropriate.

Baseline demographic and clinical characteristics were summarized for the overall study cohort. For descriptive purposes, characteristics were also presented according to whether patients received metoprolol at any time during follow-up; this patient-level classification was used only for descriptive presentation and did not define exposure status in the longitudinal analyses. Between-group comparisons were performed using Student's *t*-test or the Mann–Whitney *U* test for continuous variables and the chi-square test or Fisher’s exact test for categorical variables, as appropriate.

Standardized mean differences (SMDs) were calculated to quantify differences in baseline characteristics between patients who did and did not receive metoprolol during hospitalization. An absolute SMD <0.10 was considered indicative of minimal imbalance. The number and timing of Scr measurements, duration of follow-up, metoprolol administration frequency, and the occurrence of incident AKI were additionally summarized to characterize the longitudinal structure of the study data.

Missing data were summarized for each covariate. The handling of missing covariate values was determined according to the extent and pattern of missingness and was specified before multivariable modeling.

#### 2.6.2 Primary time-to-event analysis

The primary association between metoprolol exposure and incident AKI was evaluated using Cox proportional hazards regression with metoprolol incorporated as a time-varying exposure. Time since cohort entry was used as the underlying analysis time, and the first incident AKI event was the outcome of interest. Patients remained in the risk set until the first ascertainment of AKI or censoring at hospital discharge, in-hospital death, or the end of available follow-up.

For the primary analysis, metoprolol exposure was defined according to the prespecified 1-hospital-day-lagged daily current-use definition. Metoprolol administration on hospital day *t* determined the exposure status applied to the AKI risk interval on hospital day *t*+1. Exposure status was therefore updated throughout follow-up, and individual patients could contribute both unexposed and exposed person-time according to their daily medication administration history.

Associations were expressed as hazard ratios (HRs) with corresponding 95% confidence intervals (CIs). Covariates were selected *a priori* on the basis of their clinical relevance to both metoprolol treatment and AKI risk rather than according to statistical significance in univariable analyses. To characterize the influence of sequential confounder adjustment, five nested Cox models were fitted. Model 0 included only the lag-1 time-varying metoprolol exposure. Model 1 additionally adjusted for age, sex, ethnicity, BMI category, and baseline eGFR category. Model 2 further included diabetes mellitus, malignancy, heart failure, coronary artery disease, myocardial infarction, atrial fibrillation, and hypertension. Model 3 additionally included baseline hypotension, baseline ICU admission, and baseline sepsis. Model 4, the prespecified primary confounder-adjusted model, further included five 1-hospital-day-lagged medication covariates: renin–angiotensin system (RAS) inhibitor use, diuretic use, other β-blocker use, potentially nephrotoxic medication use, and contrast-agent exposure.

Age was modeled as a continuous variable, whereas ethnicity and BMI were modeled as categorical variables. Baseline eGFR was modeled categorically as 60–<90 and ≥90 mL min^-1^·1.73 m^-2^. Accordingly, laboratory-reported eGFR values > 200 mL min^-1^·1.73 m^-2^ were retained in the primary analysis and classified within the ≥90 mL min^-1^·1.73 m^-2^ category rather than entered into the model as extreme continuous values. Baseline Scr was not included in the primary sequential models because baseline renal function was represented by baseline eGFR; additional adjustment for baseline Scr was evaluated separately in a sensitivity analysis. Baseline covariates remained fixed throughout follow-up, whereas the five medication covariates included in Model 4 were updated at the hospital-day level and lagged by one hospital day.

Because the cohort combined data from three participating hospitals, potential between-hospital differences in patient characteristics, clinical practice, medication use, and baseline AKI risk were considered in the analytical framework. The primary estimand was defined for the pooled multicenter cohort; therefore, hospital site was not included as an additional covariate in the primary Model 4. Instead, the potential influence of the multicenter structure and heterogeneity in the metoprolol–AKI association across hospitals were evaluated in complementary site-related analyses, as described below.

To preserve temporal ordering, only covariate information available before the corresponding AKI risk interval was incorporated into the longitudinal models. Clinical information occurring after the first ascertainment of AKI did not contribute to the risk-set analysis. Acute hospital-course factors that could change during follow-up, including hypotension, ICU status, sepsis, and vasopressor use, were evaluated as lagged time-varying covariates in additional sensitivity analyses rather than incorporated into the primary Model 4.

All Cox models were stratified by calendar year to allow the baseline hazard to vary across years. Because individual patients contributed multiple start–stop risk intervals, robust variance estimates clustered at the patient level were used to account for within-patient correlation. The proportional hazards assumption was evaluated using Schoenfeld residuals and complementary graphical assessment, and multicollinearity among covariates was assessed before multivariable modeling.

All statistical tests were two-sided, and P < 0.05 was considered statistically significant. Statistical analyses were performed using R version 4.4.3 (R Foundation for Statistical Computing, Vienna, Austria). Time-varying Cox regression models were fitted using the survival package (version 3.8-3).

### 2.7 Sensitivity and secondary analyses

A series of sensitivity and secondary analyses were conducted to evaluate the robustness of the primary findings to alternative exposure definitions, concomitant nephrotoxic medication exposure, potential between-hospital differences, and assumptions regarding the temporal relationship between metoprolol administration and AKI ascertainment. Additional analyses evaluated heterogeneity across participating hospitals, whereas exploratory analyses examined the timing and dose characteristics of metoprolol exposure in relation to incident AKI. To evaluate the potential influence of unusually high laboratory-reported baseline eGFR values, an additional sensitivity analysis excluded patients with baseline eGFR >200 mL min^-1^·1.73 m^-2^ and refitted the primary Model 4 in the remaining cohort.

#### 2.7.1 Alternative exposure-lag and follow-up-time heterogeneity analyses

To assess the robustness of the primary findings to alternative exposure-lag assumptions, additional analyses were conducted using alternative hospital-day–level exposure definitions. In the primary analysis, metoprolol exposure on hospital day t was related to incident AKI first ascertained on hospital day t+1. Alternative exposure specifications included same-day exposure, in which metoprolol use on day t was related to AKI first ascertained on the same hospital day, and a two-hospital-day lag, in which metoprolol use on day t was related to AKI first ascertained on day t+2. The same-day analysis was considered particularly susceptible to reverse temporal attribution because exposure and outcome ascertainment occurred within the same hospital day.

As a prespecified analysis of temporal heterogeneity, the association between lag-1 metoprolol exposure and the first incident AKI event was evaluated across four follow-up-time intervals since cohort entry: 0 to <24 h, 24 to <48 h, 48 to <72 h, and ≥72 h. The same 1-hospital-day-lagged daily current-use exposure definition used in the primary analysis was retained within each interval. These intervals represented strata of follow-up time since cohort entry and were not alternative exposure-lag definitions.

Because metoprolol exposure was reconstructed from hospital-day–level medication execution records and exact within-day administration timestamps were not consistently available across participating hospitals, these interval-specific estimates were interpreted as follow-up-time–stratified associations under a day-level exposure proxy rather than as exact elapsed-time effects from metoprolol administration to Scr measurement or AKI ascertainment.

The estimates from the narrower early follow-up intervals were relatively imprecise. Therefore, an additional *post hoc* exploratory piecewise Cox analysis was subsequently performed using broader follow-up-time intervals of 0 to <3 days, 3 to <7 days, and ≥7 days to obtain more stable interval-specific estimates and to further characterize the overall temporal pattern of the association. These broader intervals were not prespecified, and the corresponding results were interpreted as exploratory and hypothesis-generating. The prespecified four-interval analysis remained the primary temporal-heterogeneity analysis.

#### 2.7.2 Nephrotoxic medication sensitivity analysis

Because concomitant exposure to potentially nephrotoxic medications may independently increase the risk of AKI, the primary analysis retained these patients and incorporated nephrotoxic medication exposure as a time-varying covariate. As a complementary sensitivity analysis, the primary time-varying Cox model was repeated after excluding person-time associated with prespecified potentially nephrotoxic medication exposure. The exposure definition, outcome ascertainment, follow-up structure, and covariate adjustment were otherwise maintained consistently with the primary analysis.

This analysis was performed to determine whether the association between metoprolol and incident AKI persisted when the potential influence of concomitant nephrotoxic medications was further restricted.

#### 2.7.3 Multicenter sensitivity and heterogeneity analyses

To assess the robustness of the pooled multicenter estimate to potential between-hospital differences, several complementary site-related analyses were performed using the same lag-1 time-varying metoprolol exposure definition and covariate structure as the primary Model 4. First, Model 4 was refitted with hospital site included as a fixed categorical effect to account for measured between-site differences not captured by the individual-level covariates. Second, a site-stratified Cox model was fitted, allowing the baseline hazard function to vary across hospitals while estimating a common metoprolol exposure effect across sites. Third, potential effect modification by hospital was evaluated by including a hospital site × lag-1 metoprolol exposure interaction term; the corresponding interaction test was used to assess statistical evidence of heterogeneity in the exposure–AKI association across hospitals. Finally, site-specific Cox models were fitted separately within each hospital to examine the direction and magnitude of the association at the individual-center level. Site-specific estimates were considered non-estimable when sparse exposure-related AKI events or resulting model instability prevented reliable estimation of a finite effect estimate. These site-specific analyses were considered complementary and were not used to replace the pooled multicenter primary analysis.

#### 2.7.4 Exploratory dose-related analysis

Dose-related associations were evaluated as exploratory secondary analyses. Metoprolol dose was derived from actual medication administration records rather than prescribed dose. Daily administered dose was calculated as the total amount of metoprolol actually received within each hospital day and was evaluated in relation to subsequent incident AKI using the same temporal framework as the primary analysis.

Given the limited number of patients and AKI events at higher metoprolol doses, dose was evaluated preferentially as a continuous or ordinal exposure rather than relying exclusively on sparsely populated categorical groups. Where categorical dose levels were presented for clinical interpretability, the number of exposed person-days and AKI events within each category was reported together with effect estimates and 95% CIs. Tests for trend were performed where appropriate.

Dose-related findings were interpreted as exploratory evidence of a possible exposure gradient rather than as demonstration of a biological dose–response relationship, because higher administered doses may also reflect differences in cardiovascular disease severity, treatment indication, and hemodynamic status.

#### 2.7.5 Secondary and complementary analyses

Several secondary and complementary analyses were conducted to evaluate the association between metoprolol exposure and incident AKI under alternative exposure definitions and statistical frameworks. These analyses were considered complementary to the prespecified primary lag-1 time-varying Cox analysis and were not intended to replace the primary estimand.

For patient-level fixed-exposure analyses, patients were classified as metoprolol ever-users if they received metoprolol at any time during hospitalization and as never-users otherwise. Associations with incident AKI were evaluated using cause-specific Cox regression and logistic regression. A Fine–Gray subdistribution hazards model was additionally fitted, with death before AKI treated as a competing event.

As a complementary baseline-confounding analysis, propensity scores for metoprolol ever-use were estimated using logistic regression including age, sex, baseline eGFR, baseline Scr, diabetes mellitus, malignancy, heart failure, coronary artery disease, myocardial infarction, atrial fibrillation, and hypertension. Patients were matched 1:1 using nearest-neighbor matching on the logit of the propensity score without replacement. No caliper restriction was applied in the analysis reported here. Covariate balance was assessed using standardized mean differences before and after matching.

To assess the potential influence of missing covariate data, multiple imputation was performed using the *mice* procedure. Candidate variables included age, sex, baseline eGFR, baseline Scr, BMI, major comorbidities, baseline hypotension, ICU admission, and sepsis. Because only BMI contained missing observations in the analytic dataset, BMI was the only variable requiring imputation. Five imputed datasets were generated using five iterations with a random seed of 20260829. BMI was treated as a continuous variable and imputed using predictive mean matching. The lag-1 time-varying Cox model was fitted separately in each imputed dataset, and regression coefficients and variances were combined using Rubin’s rules.

An exploratory marginal structural model was additionally fitted to address measured time-varying confounding. Stabilized inverse probability-of-treatment weights were estimated using a numerator model including age, sex, baseline eGFR, and baseline Scr. The denominator model additionally included previous-day hypotension, ICU status, sepsis, vasopressor use, RAS inhibitor use, diuretic use, nephrotoxic medication exposure, and contrast-agent exposure. Stabilized weights were truncated at the 1st and 99th percentiles, and the weighted Cox model was fitted using patient-clustered robust variance estimation. Because the validity of this approach depends on correct specification of the treatment models, adequate positivity, and measurement of relevant time-varying confounders, the marginal structural model was considered exploratory.

Finally, secondary outcome analyses evaluated AKI stage ≥2 and stage ≥3 according to KDIGO serum creatinine criteria using the same lag-1 exposure framework. Analyses based on urine-output criteria or renal replacement therapy were not performed because these data were not consistently available across the participating hospitals.

### 2.8 Subgroup analyses

Prespecified subgroup analyses were performed to explore whether the association between metoprolol exposure and incident AKI differed across clinically relevant patient characteristics. Subgroups were defined according to age, sex, baseline renal function, cardiovascular comorbidities, diabetes mellitus, medication burden, and other clinically relevant characteristics. Where appropriate and supported by sufficient sample size and event counts, additional subgroup analyses were conducted according to heart failure, coronary artery disease, and concomitant use of medications relevant to renal perfusion or AKI risk.

All subgroup analyses used the same 1-hospital-day lagged daily current-use exposure definition and time-varying Cox proportional hazards framework as the primary analysis. Within each subgroup, metoprolol current use was compared with non-current use during the corresponding risk intervals, and associations were expressed as HRs with 95% CIs. Multivariable models were adjusted using the same prespecified covariate framework as the primary analysis, excluding the variable used to define the corresponding subgroup where appropriate.

Effect modification was formally assessed by including an interaction term between time-varying metoprolol exposure and the subgroup variable in the Cox model. The *P* value for interaction was used to evaluate statistical evidence of heterogeneity across subgroup levels. Differences in statistical significance between individual subgroup-specific estimates were not interpreted as evidence of effect modification in the absence of a statistically supported interaction.

Subgroup analyses were considered exploratory. Accordingly, emphasis was placed on the direction, magnitude, precision, and consistency of subgroup-specific HR estimates and their 95% CIs, together with the corresponding interaction tests, rather than on statistical significance within individual strata. Subgroups with limited numbers of exposed person-days or AKI events were interpreted cautiously because of the potential for unstable effect estimates.

## 3 Results

### 3.1 Study population and baseline characteristics

A total of 291,049 candidate hospitalizations were initially screened, of which 284,426 involved patients aged ≥18 years. Among these, 73,934 had an available first valid laboratory-reported eGFR, while 210,492 were excluded because a valid first eGFR was unavailable. Of the 73,934 patients with an available first eGFR, 58,002 had an eGFR ≥60 mL min^-1^·1.73 m^-2^ and 15,932 with an eGFR <60 mL min^-1^·1.73 m^-2^ were excluded. After requiring the baseline serum creatinine (Scr) and eGFR measurements to be paired within 1 h, 57,969 patients remained eligible for further assessment. An additional 588 patients were excluded because of pre-existing renal or urinary tract diseases identified using the prespecified ICD-10 N00–N29 criteria. After further application of the prespecified eligibility criteria, including retention of the first eligible hospitalization per patient, availability of executable medication records, absence of AKI at or before cohort entry, and sufficient post-entry Scr follow-up, the final analytic cohort comprised 20,333 patients (Figure 1). These patients contributed 284,470 start–stop risk intervals, during which 1,050 incident AKI events were identified.

**FIGURE 1:**
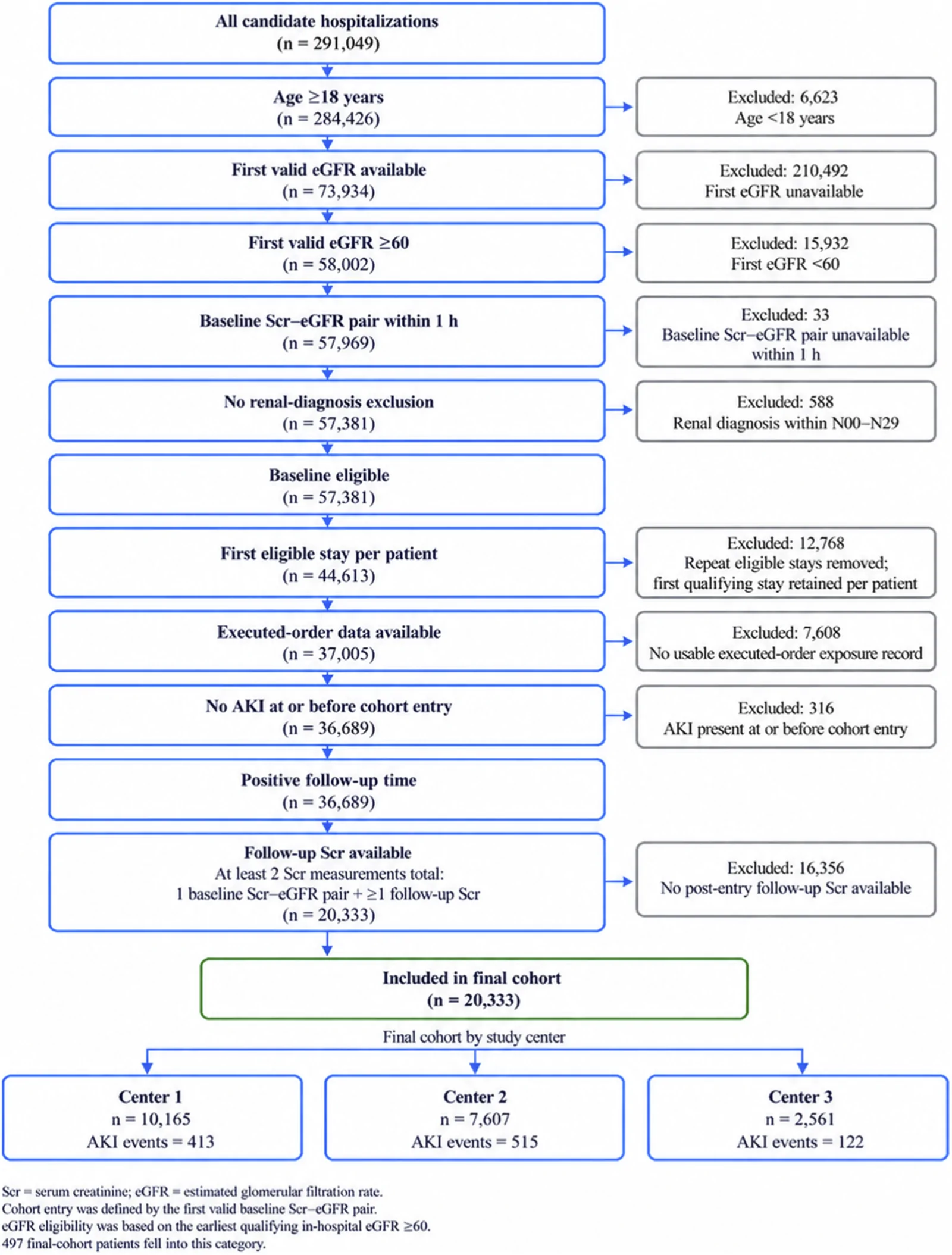
Flowchart of patient selection process.

For descriptive purposes, 2,348 patients (11.5%) received metoprolol at least once during hospitalization and 17,985 (88.5%) did not receive metoprolol during follow-up. This patient-level ever-use classification was used only to describe baseline characteristics and did not represent the exposure definition used in the longitudinal analyses. Patients who received metoprolol were generally older and had higher baseline Scr and lower baseline eGFR than those who did not receive metoprolol. Cardiovascular comorbidities, including coronary artery disease, myocardial infarction, and heart failure, as well as the use of several concomitant cardiovascular medications, were also more frequent among patients who received metoprolol (Table 1). These differences were consistent with substantial differences in the underlying clinical characteristics of patients receiving and not receiving metoprolol.

**TABLE 1:** Baseline characteristics of the study cohort according to metoprolol use during hospitalization.

Characteristic	Overall cohort (N = 20,333)	Never-use (n = 17,985)	Metoprolol ever-use (n = 2,348)	SMD
Age, years	56.30 (16.28); 57.00 [46.00, 68.00]	55.03 (16.17); 56.00 [44.00, 66.00]	66.07 (13.60); 67.00 [57.00, 76.00]	0.739
Female sex, n (%)	9054 (44.53%)	8097 (45.02%)	957 (40.76%)	−0.086
Ethnicity, n (%)				
Han	17,747 (87.28%)	15,688 (87.23%)	2,059 (87.69%)	0.014
Non-Han	2,574 (12.66%)	2,286 (12.71%)	288 (12.27%)	−0.013
Unknown/Missing	12 (0.06%)	11 (0.06%)	1 (0.04%)	−0.008
BMI category, n (%)				
Underweight	1,233 (6.06%)	1,128 (6.27%)	105 (4.47%)	−0.080
Normal weight	8,508 (41.84%)	7,748 (43.08%)	760 (32.37%)	−0.222
Overweight	4,987 (24.53%)	4,404 (24.49%)	583 (24.83%)	0.008
Obesity	1,680 (8.26%)	1,484 (8.25%)	196 (8.35%)	0.003
Unknown/Missing	3,925 (19.30%)	3,221 (17.91%)	704 (29.98%)	0.286
Baseline Scr, μmol/L	63.86 (17.67); 63.00 [53.00, 73.00]	63.44 (17.69); 62.00 [53.00, 73.00]	67.10 (17.15); 66.00 [55.00, 77.00]	0.210
Baseline eGFR, mL/min/1.73 m^2^	96.26 (65.57); 88.94 [75.52, 108.20]	97.02 (67.67); 90.09 [75.82, 108.20]	90.48 (46.01); 83.48 [71.51, 100.20]	−0.113
eGFR 60–<90, n (%)	10,310 (50.71%)	8,876 (49.35%)	1,434 (61.07%)	0.237
eGFR ≥90, n (%)	10,023 (49.29%)	9,109 (50.65%)	914 (38.93%)	−0.237
Diabetes mellitus, n (%)	456 (2.24%)	378 (2.10%)	78 (3.32%)	0.075
Malignancy, n (%)	1,494 (7.35%)	1,424 (7.92%)	70 (2.98%)	−0.219
Heart failure, n (%)	168 (0.83%)	65 (0.36%)	103 (4.39%)	0.267
Coronary artery disease, n (%)	1,526 (7.51%)	404 (2.25%)	1,122 (47.79%)	1.236
Myocardial infarction, n (%)	804 (3.95%)	171 (0.95%)	633 (26.96%)	0.810
Atrial fibrillation, n (%)	46 (0.23%)	17 (0.09%)	29 (1.24%)	0.141
Hypertension, n (%)	371 (1.82%)	298 (1.66%)	73 (3.11%)	0.095
Baseline hypotension, n (%)	776 (3.82%)	673 (3.74%)	103 (4.39%)	0.033
Baseline ICU admission, n (%)	746 (3.67%)	671 (3.73%)	75 (3.19%)	−0.029
RAS inhibitor use, n (%)	2,768 (13.61%)	1,215 (6.76%)	1,553 (66.14%)	1.568
Diuretic use, n (%)	8,231 (40.48%)	7,050 (39.20%)	1,181 (50.30%)	0.225
Other β-blocker use, n (%)	745 (3.66%)	500 (2.78%)	245 (10.43%)	0.312
Nephrotoxic medication use, n (%)	7,640 (37.57%)	6,827 (37.96%)	813 (34.63%)	−0.069
Contrast agent use, n (%)	382 (1.88%)	337 (1.87%)	45 (1.92%)	0.003

Data are presented as mean (SD) or median [IQR] for continuous variables, as appropriate, and as n (%) for categorical variables. SMDs were calculated to quantify differences in baseline characteristics between the metoprolol ever-use and never-use groups, with an absolute SMD <0.10 considered indicative of a small between-group difference. The ever-use classification was used for descriptive purposes only and did not represent the time-varying exposure definition used in the primary analysis. Comorbidities were ascertained from hospitalization-linked diagnosis records using prespecified ICD-10 code groups supplemented by diagnosis-text matching. The available diagnosis records did not consistently distinguish admission, discharge, principal, or secondary diagnosis status and were not supplemented using outpatient histories, prior hospitalization diagnoses, longitudinal problem lists, or medication proxies. Accordingly, the reported frequencies may underestimate the prevalence of chronic comorbidities in the study population.

BMI was unavailable, invalid, or classified as unknown in 3,925 patients (19.3%); these patients were retained in the primary analysis using a prespecified Unknown/Missing category. Ethnicity was classified as Han in 17,747 patients (87.28%), non-Han in 2,574 (12.66%), and Unknown/Missing in 12 (0.06%). Other core variables required for cohort entry and outcome ascertainment, including age, sex, baseline Scr, baseline eGFR, and follow-up information, were available for the final analytic cohort. Echocardiographic LVEF data were available only for a subset of patients and were therefore not included in the primary multivariable analysis.

### 3.2 Incidence of AKI and longitudinal exposure characteristics

The final cohort contributed 284,470 start–stop risk intervals during longitudinal follow-up. Metoprolol exposure was concentrated within a relatively small proportion of the cohort but, among ever-users, treatment generally extended across multiple hospital days, resulting in substantial variation in cumulative exposure and daily dose (Table 2). The number of post-entry Scr measurements was slightly higher among metoprolol ever-users, whereas the duration of follow-up was broadly comparable between the two descriptive groups.

**TABLE 2:** Longitudinal exposure characteristics and incidence of acute kidney injury during follow-up.

Characteristic	Overall	Metoprolol-unexposed	Metoprolol-exposed
Patients, n	20,333	17,985	2,348
Start–stop risk intervals, n	284,470	251,233	33,237
Hospital length of stay, days, mean (SD); median [IQR]	16.79 (15.64); 12.97 [8.16, 20.89]	16.90 (15.98); 12.99 [8.03, 21.02]	15.93 (12.74); 12.68 [9.14, 18.00]
Follow-up time, days, mean (SD); median [IQR]	13.02 (10.83); 10.11 [6.21, 16.03]	13.00 (10.83); 10.07 [6.16, 16.12]	13.12 (10.87); 10.74 [7.68, 15.27]
Scr measurements from baseline, mean (SD); median [IQR]	4.11 (3.69); 3.00 [2.00, 5.00]	4.05 (3.67); 3.00 [2.00, 5.00]	4.55 (3.87); 4.00 [2.00, 5.00]
Post-entry Scr measurements, mean (SD); median [IQR]	3.11 (3.69); 2.00 [1.00, 4.00]	3.05 (3.67); 2.00 [1.00, 4.00]	3.55 (3.87); 3.00 [1.00, 4.00]
Metoprolol exposure days, mean (SD); median [IQR]	0.96 (3.34); 0.00 [0.00, 0.00]	0.00 (0.00); 0.00 [0.00, 0.00]	8.34 (5.94); 8.00 [3.00, 12.00]
Cumulative metoprolol dose, mg, mean (SD); median [IQR]	66.64 (244.19); 0.00 [0.00, 0.00]	0.00 (0.00); 0.00 [0.00, 0.00]	577.05 (471.06); 498.75 [190.00, 855.00]
Maximum daily metoprolol dose, mg, mean (SD); median [IQR]	26.49 (108.88); 0.00 [0.00, 0.00]	0.00 (0.00); 0.00 [0.00, 0.00]	229.43 (236.90); 95.00 [47.50, 380.00]
Incident AKI, n (%)	1,050 (5.16%)	824 (4.58%)	226 (9.63%)
AKI within 24 h after cohort entry, n (%)	105 (0.52%)	74 (0.41%)	31 (1.32%)
AKI within 48 h after cohort entry, n (%)	269 (1.32%)	186 (1.03%)	83 (3.53%)
In-hospital death, n (%)	117 (0.58%)	91 (0.51%)	26 (1.11%)
Any low blood pressure during follow-up, n (%)	261 (1.28%)	226 (1.26%)	35 (1.49%)
Any ICU admission during follow-up, n (%)	1,163 (5.72%)	1,036 (5.76%)	127 (5.41%)
Any sepsis during follow-up, n (%)	48 (0.24%)	41 (0.23%)	7 (0.30%)
ACEI use, n (%)	854 (4.20%)	273 (1.52%)	581 (24.74%)
ARB use, n (%)	2057 (10.12%)	984 (5.47%)	1,073 (45.70%)
ARNI use, n (%)	862 (4.24%)	233 (1.30%)	629 (26.79%)
Direct renin inhibitor use, n (%)	0 (0.00%)	0 (0.00%)	0 (0.00%)
Diuretic use, n (%)	8231 (40.48%)	7,050 (39.20%)	1,181 (50.30%)
Vasopressor use, n (%)	1,576 (7.75%)	1,124 (6.25%)	452 (19.25%)
Other β-blocker use, n (%)	745 (3.66%)	500 (2.78%)	245 (10.43%)
Nephrotoxic medication use, n (%)	7,640 (37.57%)	6,827 (37.96%)	813 (34.63%)
Contrast-agent use, n (%)	382 (1.88%)	337 (1.87%)	45 (1.92%)

During follow-up, 1,050 patients (5.16%) developed incident AKI. The crude cumulative proportion of AKI was higher among metoprolol ever-users than among never-users, with a similar pattern observed for AKI occurring early after cohort entry. In-hospital mortality was uncommon overall but was also numerically higher among metoprolol ever-users. These patient-level comparisons were descriptive and did not account for the time-varying nature of metoprolol exposure or differences in underlying clinical characteristics.

Longitudinal clinical and medication profiles differed between the two groups. Metoprolol ever-users more frequently received cardiovascular and hemodynamically relevant therapies, particularly renin–angiotensin system–targeting agents, diuretics, vasopressors, and other β-blockers. In contrast, the proportions exposed to nephrotoxic medications, contrast agents, ICU care, hypotension, or sepsis were generally comparable or showed relatively modest absolute differences between the descriptive groups (Table 2). The relatively low observed frequency of sepsis should be interpreted in light of the source-specific ascertainment strategy, because sepsis status was based on hospital-provided sepsis-related diagnosis records rather than systematic re-screening of all hospitalization diagnoses. These longitudinal characteristics were incorporated, where prespecified, into the subsequent time-varying analyses rather than interpreted as unadjusted between-group effects.

### 3.3 Primary lag-1 time-varying Cox analysis

In the unadjusted time-varying Cox model, lag-1 metoprolol exposure was associated with a higher hazard of incident AKI (HR, 2.53; 95% CI, 2.13–3.00; P < 0.001). After adjustment for demographic characteristics and baseline renal function, the association was attenuated but remained statistically significant (HR, 2.01; 95% CI, 1.68–2.41; P < 0.001). Further adjustment for prespecified comorbidities reduced the HR to 1.31 (95% CI, 1.03–1.67; P = 0.028). After additional adjustment for baseline severity-related factors, the HR was 1.43 (95% CI, 1.12–1.82; P = 0.004). In the primary confounder-adjusted model, which further accounted for major medication-related confounders, lag-1 metoprolol exposure remained associated with a higher hazard of incident AKI (HR, 1.37; 95% CI, 1.05–1.79; P = 0.022) (Table 3).

**TABLE 3:** Association between lag-1 time-varying metoprolol exposure and incident AKI in sequential Cox regression models.

Model	Adjustment stage	HR (95% CI)	P	Risk intervals/AKI events
Model 0	Unadjusted	2.53 (2.13–3.00)	<0.001	284,470/1,050
Model 1	Demographic characteristics + baseline renal function	2.01 (1.68–2.41)	<0.001	284,470/1,050
Model 2	Model 1 + comorbidities	1.31 (1.03–1.67)	0.028	284,470/1,050
Model 3	Model 2 + baseline severity-related factors	1.43 (1.12–1.82)	0.004	284,470/1,050
Model 4	Model 3 + major medication-related confounders	1.37 (1.05–1.79)	0.022	284,470/1,050

Abbreviations: AKI, acute kidney injury; CI, confidence interval; HR, hazard ratio.

Model 0 included only the lag-1, time-varying metoprolol exposure. Model 1 additionally adjusted for age, sex, ethnicity, BMI, category, and baseline eGFR, category. Model 2 further adjusted for diabetes mellitus, malignancy, heart failure, coronary artery disease, myocardial infarction, atrial fibrillation, and hypertension. Model 3 additionally adjusted for baseline hypotension, baseline ICU, admission, and baseline sepsis. Model 4 additionally included five 1-hospital-day-lagged medication covariates: RAS, inhibitor use, diuretic use, other β-blocker use, potentially nephrotoxic medication use, and contrast-agent exposure. Age was modeled as a continuous variable; ethnicity, BMI, and baseline eGFR, were modeled as categorical variables. All models were stratified by calendar year and used patient-clustered robust variance estimates. Model 4 was the prespecified primary confounder-adjusted model. All models included 284,470 start–stop risk intervals and 1,050 incident AKI, events. Baseline Scr was not included in the primary models and was evaluated separately in a sensitivity analysis.

The progressive attenuation of the effect estimate from the crude model (HR, 2.53) to the primary confounder-adjusted model (HR, 1.37) indicated that a substantial proportion of the crude association was accounted for by measured patient characteristics and clinical confounders. Nevertheless, the association remained statistically significant after the prespecified sequential adjustment.

### 3.4 Multicenter sensitivity and heterogeneity analyses

Multicenter sensitivity analyses yielded estimates closely aligned with the primary Model 4 (Table 4). Inclusion of hospital site as a fixed categorical effect and stratification of the Cox baseline hazard by hospital site produced effect estimates that were essentially unchanged from the pooled primary analysis. No statistical evidence of heterogeneity in the metoprolol–AKI association across hospitals was detected (P for interaction = 0.844). In site-specific analyses, the estimate for Center 1 was directionally consistent with the pooled analysis but imprecise (HR, 1.42; 95% CI, 0.95–2.13; P = 0.087). Stable site-specific estimates could not be obtained for Centers 2 and 3 and were therefore reported as not estimable.

**TABLE 4:** Multicenter sensitivity and heterogeneity analyses of the association between lag-1 time-varying metoprolol exposure and incident AKI.

Analysis/Center	Patients, n	Exposure-related/total AKI events, n	HR (95% CI)	P-value	P for interaction
Primary Model 4, pooled cohort	20,333	—/1,050	1.37 (1.05–1.79)	0.022	—
Model 4 + fixed site effect	20,333	—/1,050	1.36 (1.04–1.79)	0.024	—
Model 4 stratified by site	20,333	—/1,050	1.37 (1.05–1.79)	0.022	—
Center 1, site-specific model	10,165	62/413	1.42 (0.95–2.13)	0.087	—
Center 2, site-specific model	7,607	90/515	NE	—	—
Center 3, site-specific model	2,561	9/122	NE	—	—
Site × metoprolol exposure interaction	—	—	—	—	0.844

Abbreviations: AKI, acute kidney injury; CI, confidence interval; HR, hazard ratio; NE, not estimable.

All site-related analyses used the same lag-1, time-varying metoprolol exposure definition and covariate structure as the primary Model 4 unless otherwise specified. The fixed-site model included hospital site as a categorical covariate. The site-stratified model allowed the baseline hazard function to vary across hospitals while estimating a common metoprolol exposure effect. The interaction P value was derived from the hospital site × lag-1, metoprolol exposure interaction model. Site-specific models were fitted separately within each hospital. NE, indicates that a stable finite effect estimate could not be reliably obtained.

### 3.5 Proportional hazards assessment and temporal heterogeneity

The proportional hazards assumption of the primary adjusted model was evaluated using Schoenfeld residuals. No statistically significant evidence of non-proportionality was detected for the lag-1 metoprolol exposure term (P = 0.053), although the global Schoenfeld test indicated evidence of non-proportionality for the overall Model 4 (P < 0.001).

In the prespecified temporal-heterogeneity analysis, the association between lag-1 metoprolol exposure and incident AKI was evaluated across four follow-up-time intervals since cohort entry. The adjusted HR was 1.60 (95% CI, 1.03–2.49; P = 0.038) during 0–<24 h, 2.75 (95% CI, 1.81–4.19; P < 0.001) during 24–<48 h, 2.59 (95% CI, 1.51–4.46; P < 0.001) during 48–<72 h, and 0.99 (95% CI, 0.71–1.37; P = 0.942) at ≥72 h (Table 5). Overall, the prespecified analysis suggested that the association varied across follow-up time, with elevated estimates during the first 72 h after cohort entry and an estimate close to the null thereafter. These interval-specific estimates reflect follow-up-time strata under the hospital-day–level exposure reconstruction and should not be interpreted as exact elapsed-time effects from metoprolol administration to AKI ascertainment.

**TABLE 5:** Proportional hazards assessment and temporal heterogeneity in the association between lag-1 metoprolol exposure and incident AKI.

Analysis	Follow-up interval/test	HR (95% CI)	P value
Panel A. proportional hazards assessment			
Schoenfeld residual test	Metoprolol exposure	—	0.053
​	Global test	—	<0.001
Panel B. prespecified temporal-heterogeneity analysis			
Prespecified follow-up-time intervals	0–<24 h	1.60 (1.03–2.49)	0.038
​	24–<48 h	2.75 (1.81–4.19)	<0.001
​	48–<72 h	2.59 (1.51–4.46)	<0.001
​	≥72 h	0.99 (0.71–1.37)	0.942
Panel C. post hoc exploratory broader-window analysis			
Post hoc follow-up-time intervals	0–<3 days	2.19 (1.58–3.02)	<0.001
​	3–<7 days	1.07 (0.70–1.62)	0.760
​	≥7 days	0.91 (0.61–1.37)	0.667
Likelihood-ratio comparison^†^	Constant-HR model vs. *post hoc* broader-window piecewise model	—	<0.001

Abbreviations: AKI, acute kidney injury; CI, confidence interval; HR, hazard ratio.

Panel A presents the proportional hazards assessment of the primary adjusted Cox model using Schoenfeld residuals. Panel B presents the prespecified temporal-heterogeneity analysis across four follow-up-time intervals since cohort entry (0–<24 h, 24–<48 h, 48–<72 h, and ≥72 h). Panel C presents the *post hoc* exploratory analysis using broader follow-up-time intervals (0–<3 days, 3–<7 days, and ≥7 days), which was subsequently performed to improve the precision of interval-specific estimates. In both temporal-heterogeneity analyses, metoprolol exposure retained the same 1-hospital-day-lagged daily current-use definition as in the primary analysis; therefore, the intervals represent strata of follow-up time since cohort entry rather than alternative exposure-lag definitions. Because exact within-day administration timestamps were not consistently available, the interval-specific estimates should not be interpreted as exact elapsed-time effects from metoprolol administration to AKI, ascertainment.

^†^ The likelihood-ratio test compares the *post hoc* broader-window piecewise model in Panel C with the constant-HR, primary model; it does not apply to the prespecified four-interval analysis in Panel B.

Because the estimates from the narrower early follow-up intervals were relatively imprecise, an additional *post hoc* exploratory analysis using broader follow-up intervals was performed. The HR was 2.19 (95% CI, 1.58–3.02; P < 0.001) during 0–<3 days, 1.07 (95% CI, 0.70–1.62; P = 0.760) during 3–<7 days, and 0.91 (95% CI, 0.61–1.37; P = 0.667) at ≥7 days (Table 5, Panel C). The *post hoc* broader-window piecewise model provided a better fit than the constant-effect model (likelihood-ratio statistic, 21.00; df = 2; P < 0.001). Although this broader-window analysis showed a temporal pattern consistent with the prespecified analysis, it was considered exploratory because the intervals were defined after the prespecified analysis.

The constant-HR lag-1 Model 4 remained the prespecified primary association model, whereas the four-interval analysis represented the prespecified assessment of temporal heterogeneity and the broader three-interval analysis was considered *post hoc* and exploratory.

### 3.6 Sensitivity analyses

Sensitivity analyses generally yielded effect estimates in the same direction as the primary analysis, although the magnitude and statistical precision varied across specifications (Table 6). After excluding AKI events occurring within 24 h or 48 h of cohort entry, the adjusted HRs were 1.51 (95% CI, 1.15–1.99; P = 0.003) and 1.53 (95% CI, 1.12–2.07; P = 0.007), respectively. The same-day exposure analysis yielded a similar positive association (HR, 1.43; 95% CI, 1.10–1.86; P = 0.007).

**TABLE 6:** Sensitivity analyses of the association between metoprolol exposure and incident AKI.

Sensitivity analysis	Patients, n	AKI events, n	HR (95% CI)	P-value
Primary analysis*	20,333	1,050	1.37 (1.05–1.79)	0.022
Excluding AKI within 24 h	20,228	945	1.51 (1.15–1.99)	0.003
Excluding AKI within 48 h	20,064	781	1.53 (1.12–2.07)	0.007
Same-day exposure	20,333	1,050	1.43 (1.10–1.86)	0.007
Two-day lagged exposure	20,101	895	1.27 (0.96–1.68)	0.090
Additional adjustment for ICU status	20,333	1,050	1.40 (1.07–1.84)	0.015
Additional adjustment for sepsis status	20,333	1,050	NE	—
Additional adjustment for vasopressor use	20,333	1,050	1.29 (0.98–1.70)	0.070
Additional adjustment for lagged low blood pressure	20,333	1,050	1.37 (1.04–1.79)	0.023
Joint adjustment for acute illness severity†	20,333	1,050	NE	—
Additional adjustment for Scr monitoring frequency	20,333	1,050	1.35 (1.04–1.76)	0.024
BMI complete-case analysis	16,408	774	1.53 (1.10–2.14)	0.013
Excluding baseline eGFR >200 mL min^-1^·1.73 m^-2^	20,124	1,014	1.38 (1.05–1.82)	0.019
≥2 follow-up Scr measurements	11,982	796	1.42 (1.05–1.94)	0.024

Abbreviations: AKI, acute kidney injury; BMI, body mass index; CI, confidence interval; eGFR, estimated glomerular filtration rate; HR, hazard ratio; ICU, intensive care unit; NE, not estimable; Scr, serum creatinine.

* Primary analysis refers to the fully adjusted lag-1, time-varying Cox model reported in Table 3.

^†^ The joint acute illness severity model included ICU, status, sepsis, vasopressor use, and lagged low blood pressure. A stable finite estimate could not be obtained because of complete or quasi-complete separation. NE, not estimable. Same-day exposure was reconstructed at the hospital-day level and did not represent exact administration-to-AKI, elapsed time.

The association remained statistically significant after additional adjustment for Scr monitoring frequency and in analyses restricted according to BMI completeness, baseline eGFR, and the availability of repeated follow-up Scr measurements (Table 6). In contrast, the estimate was attenuated with a 2-hospital-day exposure lag (HR, 1.27; 95% CI, 0.96–1.68; P = 0.090) and after additional adjustment for vasopressor use (HR, 1.29; 95% CI, 0.98–1.70; P = 0.070). Additional adjustment for ICU status or lagged low blood pressure yielded estimates comparable to the primary analysis, whereas the sepsis component model and the joint acute illness severity model were not estimable because of model instability (Table 6).

### 3.7 Dose- and administration-related analyses

Dose-related analyses did not demonstrate a consistent monotonic dose–response relationship (Table 7). When daily metoprolol dose was modeled continuously, no significant linear association was observed per 25-mg increase (HR, 1.01; 95% CI, 0.98–1.04; P = 0.632). In the categorical analysis, the 0–<50 mg/day category comprised 14,851 exposed person-days and 133 AKI events (HR, 1.34; 95% CI, 1.02–1.77; P = 0.039), whereas the 50–100 mg/day category comprised 1,833 exposed person-days and 27 AKI events (HR, 1.89; 95% CI, 1.20–2.96; P = 0.006). The >100 mg/day category comprised 678 exposed person-days but only one AKI event, resulting in a highly imprecise estimate (HR, 0.25; 95% CI, 0.03–1.77; P = 0.165). Although the dose-restricted analysis at ≤200 mg/day showed an association per 25-mg increase (HR, 1.14; 95% CI, 1.04–1.26; P = 0.006), the overall findings did not support a consistent monotonic dose–response relationship. Administration-frequency analyses similarly yielded HRs of 1.32 (95% CI, 1.00–1.75; P = 0.050) for once-daily administration and 1.63 (95% CI, 1.07–2.47; P = 0.022) for administration ≥2 times daily.

**TABLE 7:** Exploratory dose- and administration-related analyses of metoprolol exposure and incident AKI.

Analysis	Exposure category/increment	Exposed person-days, n	AKI events, n	HR (95% CI)	P-value
Daily dose, continuous	Per 25-mg increase	—	—	1.01 (0.98–1.04)	0.632
Daily dose, categorical	0–<50 mg/day	14,851	133	1.34 (1.02–1.77)	0.039
​	50–100 mg/day	1,833	27	1.89 (1.20–2.96)	0.006
​	>100 mg/day	678	1	0.25 (0.03–1.77)	0.165
Dose-restricted analysis	Per 25-mg increase, ≤200 mg/day	—	—	1.14 (1.04–1.26)	0.006
Administration frequency	Once daily	14,977	131	1.32 (1.00–1.75)	0.050
​	≥2 times daily	2,387	30	1.63 (1.07–2.47)	0.022

Abbreviations: AKI, acute kidney injury; CI, confidence interval; HR, hazard ratio.

Dose-related analyses were exploratory. Daily dose was derived from actual metoprolol administration records and evaluated using the same lag-1, temporal framework as the primary analysis. Exposed person-days and AKI, events are reported for categorical dose and administration-frequency analyses. The >100 mg/day category contained only one AKI, event; therefore, the corresponding effect estimate was highly imprecise and should not be interpreted as evidence of a protective association. The overall pattern did not demonstrate a consistent monotonic dose–response relationship.

### 3.8 Subgroup analyses

Prespecified subgroup analyses were performed to explore potential heterogeneity in the association between lag-1 time-varying metoprolol exposure and incident AKI (Figure 2). Stable subgroup-specific effect estimates could be obtained for nine subgroup levels, whereas estimates for several other strata were not considered reliable because of sparse exposure–event combinations or model instability.

**FIGURE 2:**
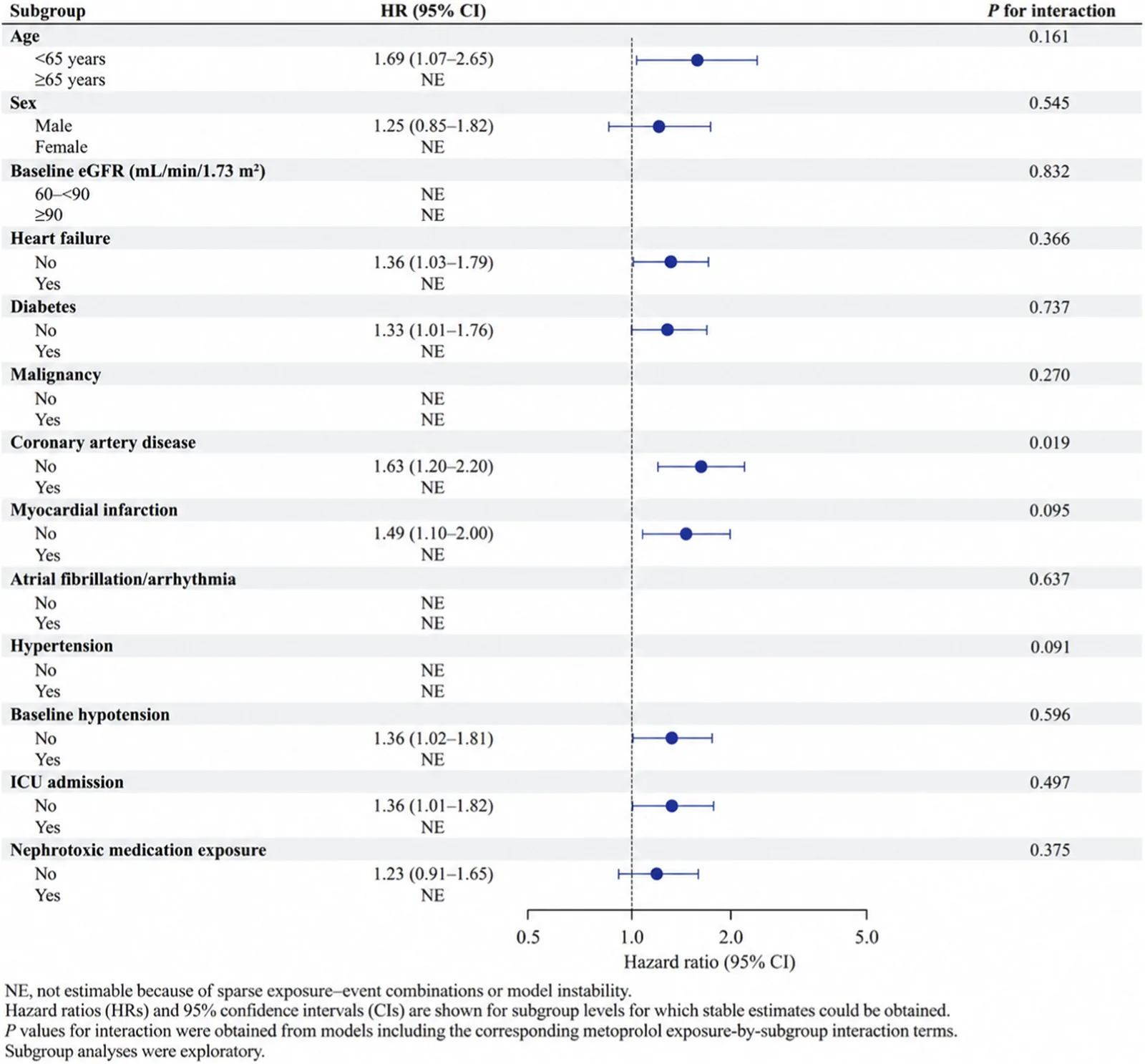
Subgroup analyses of the association between lag-1 time-varying metoprolol exposure and incident acute kidney injury.

Among the evaluable strata, the estimated HRs were generally above unity, although the magnitude and precision of the associations varied across patient characteristics. Formal interaction testing was completed for all prespecified subgroup variables. Evidence of heterogeneity was observed for coronary artery disease (*P* for interaction = 0.019), whereas no statistically significant interaction was detected for age, sex, baseline eGFR, heart failure, diabetes, malignancy, myocardial infarction, atrial fibrillation, hypertension, baseline hypotension, ICU admission, or nephrotoxic medication exposure.

Because the subgroup-specific estimate among patients with coronary artery disease could not be reliably estimated and several other subgroup strata were also not estimable, the subgroup findings were considered exploratory and interpreted cautiously.

### 3.9 Secondary and complementary analyses

Secondary and complementary analyses using alternative exposure definitions and statistical frameworks yielded effect estimates that were generally above unity, although their magnitude and precision varied across analyses (Table 8). Under the fixed ever-use exposure definition, cause-specific Cox regression (HR, 1.24; 95% CI, 1.01–1.51; P = 0.038), Fine–Gray competing-risk analysis (sHR, 1.24; 95% CI, 1.01–1.51; P = 0.038), logistic regression (OR, 1.45; 95% CI, 1.19–1.78; P < 0.001), and the propensity score–matched analysis (OR, 1.28; 95% CI, 1.05–1.57; P = 0.017) all yielded estimates above unity. Accounting for death as a competing event did not materially alter the corresponding time-to-event estimate.

**TABLE 8:** Secondary and complementary analyses of the association between metoprolol exposure and AKI.

Analysis	Exposure/outcome	Effect estimate (95% CI)	P-value
Cause-specific Cox regression	Fixed ever-use/incident AKI	HR 1.24 (1.01–1.51)	0.038
Fine–Gray competing-risk model	Fixed ever-use/incident AKI	sHR 1.24 (1.01–1.51)	0.038
Logistic regression	Fixed ever-use/incident AKI	OR 1.45 (1.19–1.78)	<0.001
Propensity score–matched analysis	Fixed ever-use/incident AKI	OR 1.28 (1.05–1.57)	0.017
Multiple-imputation analysis	Lag-1 time-varying exposure/incident AKI	HR 1.28 (0.98–1.68)	0.073
Marginal structural model†	Time-varying exposure/incident AKI	HR 2.17 (1.68–2.80)	<0.001
Severe AKI analysis	Lag-1 exposure/AKI stage ≥2	HR 1.73 (0.85–3.52)	0.128
Severe AKI analysis	Lag-1 exposure/AKI stage ≥3	HR 2.19 (0.68–7.10)	0.190

Abbreviations: AKI, acute kidney injury; CI, confidence interval; HR, hazard ratio; OR, odds ratio; sHR, subdistribution hazard ratio.

Fixed ever-use analyses estimated a different exposure contrast from the primary lag-1, time-varying analysis. PSM, used 1:1 nearest-neighbor matching without replacement and without a caliper. Multiple imputation primarily addressed missing BMI, data, with estimates pooled using Rubin’s rules. The exploratory MSM, used stabilized IPTW, truncated at the 1st and 99th percentiles. Death before AKI, was treated as a competing event in the Fine–Gray model.

^†^ The marginal structural model was exploratory and relied on the available measured time-varying covariates.

In the multiple-imputation analysis, in which missing BMI values were imputed and the primary lag-1 time-varying Cox model was refitted across the imputed datasets, the pooled estimate remained above unity but was less precise and was not statistically significant (HR, 1.28; 95% CI, 0.98–1.68; P = 0.073). The exploratory marginal structural model using stabilized inverse probability-of-treatment weighting yielded a larger estimate (HR, 2.17; 95% CI, 1.68–2.80; P < 0.001). Because the fixed-exposure analyses, multiple-imputation analysis, and marginal structural model differed in exposure definition, estimand, covariate handling, and modeling assumptions, these estimates were considered complementary rather than directly interchangeable with the prespecified primary lag-1 time-varying Cox estimate.

For more severe AKI outcomes, the point estimates remained above unity but were imprecise and not statistically significant for both AKI stage ≥2 (HR, 1.73; 95% CI, 0.85–3.52; P = 0.128) and stage ≥3 (HR, 2.19; 95% CI, 0.68–7.10; P = 0.190) (Table 8). Analyses based on urine-output criteria or renal replacement therapy were not performed because the required data were unavailable.

## 4 Discussion

In this multicenter retrospective longitudinal cohort study, metoprolol exposure was associated with a modestly higher hazard of incident AKI when modeled as a 1-hospital-day-lagged time-varying exposure. The crude association was progressively attenuated after adjustment for demographic characteristics, baseline renal function, cardiovascular and other comorbidities, severity-related factors, and medication-related confounders, with an adjusted HR of 1.37 (95% CI, 1.05–1.79). Prespecified follow-up-time analyses suggested temporal heterogeneity, with elevated associations during the first 72 h after cohort entry and an estimate close to the null thereafter; a *post hoc* analysis using broader follow-up intervals showed a compatible early-follow-up pattern. Sensitivity and complementary analyses showed partial consistency but also important model dependence: although several estimates were broadly comparable with the primary analysis, the association was attenuated and no longer statistically significant under some clinically relevant alternative specifications, including the 2-hospital-day lag analysis and additional adjustment for vasopressor use. Collectively, these findings support a modest short-term association between recent metoprolol exposure and AKI ascertainment in the primary analysis, but the substantial attenuation after adjustment and sensitivity to alternative temporal and confounding specifications indicate that the primary estimate should not be regarded as uniformly robust and argue against interpreting the association as evidence of direct nephrotoxicity.

A major methodological distinction between the present analysis and the initial analytical framework concerns the representation of exposure and time. The earlier approach primarily classified metoprolol exposure at the patient level and used propensity score matching (PSM) to address measured baseline differences between exposed and unexposed patients. Although PSM can improve comparability with respect to measured baseline covariates, a fixed patient-level exposure definition is less suited to inpatient treatment that may be initiated, discontinued, or modified during hospitalization. Such classification may inadequately distinguish treatment preceding renal deterioration from treatment initiated or continued in response to evolving cardiovascular or hemodynamic instability, thereby increasing susceptibility to reverse temporal attribution and time-related exposure misclassification. Moreover, baseline PSM does not, by itself, account for changes in disease severity, hemodynamic status, or concomitant treatment during follow-up.

The revised analytical framework was designed to address these temporal limitations. Metoprolol exposure was reconstructed from hospital-day–level medication execution records and treated as a time-varying current-use exposure, with exposure on hospital day *t* related to AKI risk on day *t*+1. Individual patients could therefore contribute both unexposed and exposed person-time, while time-varying clinical and medication-related covariates were aligned with the corresponding risk intervals. Cohort entry was anchored to an observed baseline Scr measurement with a corresponding laboratory-reported eGFR ≥60 mL min^-1^·1.73 m^-2^, and subsequent Scr measurements were evaluated sequentially using rolling KDIGO-based criteria. The principal advantage of this revised framework is therefore not merely additional statistical adjustment, but improved preservation of the temporal relationship among changing treatment exposure, evolving clinical status, and subsequent AKI ascertainment. This design more closely reflects the dynamic nature of inpatient medication use and AKI development than the original fixed-exposure framework.

The attenuation of the effect estimate under the revised framework is itself informative. Metoprolol ever-users had substantially greater cardiovascular disease burden and more frequent use of cardiovascular medications than never-users. The reduction from a strong crude association to an adjusted HR of 1.37 therefore suggests that measured patient characteristics, treatment indication, cardiovascular disease burden, and concomitant therapy explain an important component of the unadjusted association. Importantly, however, the association did not disappear after adjustment. In complementary analyses using a fixed ever-use exposure definition, cause-specific Cox, competing-risk, logistic, and PSM analyses all yielded estimates above unity, with the PSM analysis yielding an OR of 1.28 (95% CI, 1.05–1.57). Although these estimates should not be directly compared numerically with the primary time-varying HR because they represent different exposure contrasts and estimands, their direction suggests that the observed safety signal was not confined to a single analytical framework. At the same time, the more modest estimates obtained under the revised analyses demonstrate that its magnitude is sensitive to exposure definition, temporal structure, and confounding control. This interpretation is consistent with the substantial baseline differences between metoprolol ever-users and never-users documented in the current cohort.

The temporal pattern of the association provides additional insight but requires cautious interpretation. The Schoenfeld residual test did not show statistically significant evidence of non-proportionality for the lag-1 metoprolol exposure term (P = 0.053), although the global test indicated non-proportionality of the overall adjusted model. In the prespecified temporal-heterogeneity analysis, the association was elevated across the first three follow-up intervals (0–<24 h, 24–<48 h, and 48–<72 h) and was close to the null at ≥72 h, suggesting that the observed association was concentrated during early follow-up. A subsequently conducted *post hoc* analysis using broader follow-up intervals yielded a compatible pattern, with an elevated estimate during the first 3 days but not during later follow-up; the broader-window piecewise model also provided a better fit than the constant-effect model. Because these broader intervals were defined after the prespecified analysis, however, the corresponding findings should be regarded as exploratory rather than confirmatory.

A short-term hemodynamic contribution is biologically plausible in susceptible patients because β-blockade can influence heart rate, cardiac output, and systemic perfusion (Park et al., 2025), and renal dysfunction has been described as a component of BRASH syndrome in patients receiving atrioventricular nodal blocking agents (Shah et al., 2022; Majeed et al., 2023; Yan et al., 2024). However, neither the prespecified nor the *post hoc* temporal analyses establish a specific biological latency or causal mechanism. Early metoprolol exposure may instead identify patients with greater cardiovascular instability, treatment intensity, or evolving illness severity. Moreover, because exposure was reconstructed at the hospital-day level, the predefined follow-up intervals should not be interpreted as exact administration-to-AKI elapsed-time windows. The observed pattern therefore supports temporal heterogeneity in the association but does not establish an acute nephrotoxic effect of metoprolol.

Alternative exposure-lag analyses further reinforce the need for caution when interpreting the observed temporal pattern. Excluding AKI events detected within 24 or 48 h after cohort entry did not eliminate the association, making it less likely that the entire finding was attributable to renal dysfunction already detectable at cohort entry. The same-day analysis also yielded an estimate above unity, whereas the association was attenuated and no longer statistically significant with a 2-hospital-day lag. Thus, the estimated association remains sensitive to the temporal window relating exposure to AKI. Because exact within-day administration timestamps were not consistently available, the 1-day lag should be regarded as an epidemiological strategy to improve temporal separation rather than a pharmacologically defined induction period. These findings therefore strengthen temporal ordering relative to the original exposure definition but do not establish the precise interval between metoprolol administration and biological renal injury.

The dose-related findings provide little evidence for a simple monotonic dose–response relationship. No significant linear association was observed across the full dose range, categorical estimates were heterogeneous, and the highest-dose category did not show a corresponding increase despite positive findings for the intermediate-dose category and the analysis restricted to doses ≤200 mg/day. Spline modeling also suggested non-linearity. Administration at least twice daily was associated with a higher estimated hazard, whereas once-daily administration was not. These findings may reflect treatment indication, cardiovascular disease severity, treatment intensity, or sparse exposure–event combinations rather than pharmacological dose dependence. The absence of a consistent monotonic gradient therefore limits an interpretation based on direct dose-dependent nephrotoxicity.

Sensitivity analyses showed both partial consistency and important model dependence. Adjustment for Scr monitoring frequency, restriction according to BMI completeness and baseline eGFR, restriction to patients with repeated follow-up Scr measurements, and adjustment for ICU status or lagged low blood pressure generally produced estimates comparable with the primary analysis. In contrast, the 2-hospital-day lag analysis and additional adjustment for vasopressor use attenuated the association, while some models incorporating acute illness-severity factors could not yield stable estimates because of sparse exposure–event combinations, separation, or model instability. These findings indicate that the magnitude and statistical significance of the observed association were sensitive to clinically relevant assumptions regarding exposure timing and adjustment for acute hemodynamic severity, reinforcing the possibility of residual confounding by evolving illness severity and treatment intensity. The multicenter structure of the cohort was also examined in additional sensitivity analyses. Incorporating hospital site as a fixed effect or allowing the baseline hazard to vary across hospitals produced estimates closely aligned with the primary Model 4, and no statistical evidence of heterogeneity in the metoprolol–AKI association by hospital site was detected. These findings suggest that adjustment for site-level differences captured by hospital membership did not materially alter the pooled estimate. However, stable site-specific estimates could not be obtained for two of the three hospitals. Accordingly, the absence of statistically detectable site heterogeneity should not be interpreted as evidence of identical associations across centers, and between-hospital heterogeneity could not be estimated precisely. Complementary analyses further demonstrated model dependence. Multiple imputation, which primarily addressed missing BMI values, yielded an HR of 1.28 (95% CI, 0.98–1.68), whereas the exploratory marginal structural model using stabilized inverse probability-of-treatment weighting yielded a larger estimate (HR, 2.17; 95% CI, 1.68–2.80). Because the MSM depends on additional assumptions regarding model specification, positivity, and measurement of time-varying confounders, its larger estimate should not be interpreted as evidence that the primary model underestimated a causal effect. Rather, differences among the primary, fixed-exposure, multiple-imputation, and MSM analyses indicate that the magnitude of the observed association remains dependent on modeling assumptions and available confounder information.

For severe AKI, point estimates for stages ≥2 and ≥3 remained above unity but were imprecise and not statistically significant. The limited number of severe events therefore precluded firm conclusions regarding progression to more severe kidney injury. Because urine-output and renal replacement therapy data were not consistently available, these analyses were restricted to serum creatinine–based outcomes.

From a clinical perspective, the findings are best interpreted as a potential drug-safety and risk-monitoring signal rather than evidence that metoprolol is an established nephrotoxic agent. The substantially attenuated estimate after temporal reconstruction and multivariable confounder adjustment, together with the loss of statistical significance under some clinically relevant alternative specifications, suggests that the observed association may reflect a complex interplay among patient susceptibility, cardiovascular disease severity, hemodynamic instability, concomitant treatment, and other residual confounding rather than a direct nephrotoxic effect of metoprolol. In particular, attenuation in the 2-hospital-day lag analysis and after additional adjustment for vasopressor use indicates that the magnitude and statistical significance of the association are sensitive to assumptions regarding exposure timing and control of acute illness severity. Accordingly, the present findings do not establish a causal relationship between metoprolol and AKI and do not support avoidance, discontinuation, or modification of clinically indicated metoprolol therapy solely on the basis of AKI risk. Rather, they support continued attention to renal function and evolving hemodynamic and clinical status in hospitalized patients receiving metoprolol who already have other risk factors for AKI, while emphasizing the need for further studies with higher-resolution exposure, hemodynamic, and renal-function data to determine whether the observed association is causal.

Several limitations should be considered. First, residual confounding remains a major concern. Metoprolol is preferentially prescribed to patients with cardiovascular diseases that may themselves predispose to AKI. Although major cardiovascular diagnoses, hemodynamic factors, and relevant concomitant medications were incorporated into the analyses, detailed measures of disease severity, including LVEF, heart failure functional status, cardiac output, fluid balance, duration of hypotension, and indications for metoprolol treatment, were not consistently available. Thus, confounding by indication and other unmeasured or incompletely measured clinical factors cannot be excluded. In addition, ascertainment of chronic comorbidities relied on the available hospitalization-linked diagnosis records and was not supplemented using outpatient medical histories, longitudinal problem lists, medication proxies, or diagnoses from prior hospitalizations. Chronic conditions that were not recorded in these hospitalization-linked diagnosis files may therefore have been missed. This restricted ascertainment likely contributed to the unexpectedly low observed frequencies of some comorbidities and may have resulted in covariate misclassification and residual confounding. Second, exposure was reconstructed at the hospital-day level because reliable exact within-day administration timestamps were not consistently available. Medication execution records provide stronger evidence of exposure than prescription orders, but do not permit reconstruction of exact administration-to-AKI intervals. The 1-hospital-day lag therefore improves temporal ordering but cannot completely eliminate reverse temporal attribution and should not be interpreted as a pharmacologically defined induction period. Third, AKI ascertainment and cohort selection may affect outcome detection and generalizability. AKI was identified using serial Scr measurements without the urine-output criterion, and clinically determined Scr testing frequency may have introduced differential detection. Although analyses accounting for Scr monitoring yielded broadly similar estimates, residual surveillance bias cannot be excluded. Moreover, eligibility required a baseline eGFR ≥60 mL min^-1^·1.73 m^-2^ and sufficient longitudinal renal-function and medication data. The findings therefore primarily apply to hospitalized patients with preserved baseline renal function and should not be directly extrapolated to patients with pre-existing renal impairment. Finally, several secondary analyses were limited by sparse exposure–event combinations and should be considered exploratory. The broader follow-up-time intervals used in the additional piecewise analysis were defined *post hoc* after inspection of the prespecified interval-specific estimates and therefore should be interpreted as hypothesis-generating. Some models incorporating acute illness-severity factors could not be reliably estimated because of sparse exposure–event combinations, separation, or model instability, limiting our ability to fully evaluate residual confounding by evolving hemodynamic and clinical severity. Similarly, although adjustment for hospital site and stratification by site produced estimates closely aligned with the primary model and no statistical evidence of a site-by-exposure interaction was detected, stable site-specific estimates could not be obtained for two hospitals, limiting precise assessment of between-center heterogeneity. In addition, estimates for severe AKI were imprecise, and the dose analyses did not demonstrate a consistent monotonic dose–response relationship.

Despite these limitations, the study has several strengths. It included a large multicenter inpatient cohort and explicitly evaluated the potential influence of between-hospital differences on the pooled association. Exposure ascertainment was based on medication execution rather than prescription records, metoprolol exposure was reconstructed longitudinally, and patients were allowed to change exposure status during follow-up. Exposure and time-varying covariates were temporally aligned with subsequent AKI risk intervals, and first incident AKI was ascertained using rolling serum-creatinine criteria. Most importantly, the analytical redesign directly addressed key temporal limitations of the earlier fixed-exposure/PSM framework rather than relying solely on additional covariate adjustment. The resulting effect estimate was substantially more modest and clinically plausible, but a statistically significant association persisted in the prespecified primary analysis and estimates remained directionally consistent across several complementary approaches. Overall, the revised analysis identifies a substantially attenuated but persistent association between recent metoprolol exposure and incident AKI, supporting further investigation of this potential short-term safety signal using higher-resolution exposure, cardiovascular, and hemodynamic data.

## 5 Conclusion

In this multicenter retrospective longitudinal cohort study, lag-1 time-varying metoprolol exposure was associated with a modestly increased hazard of incident AKI in the primary adjusted analysis. Prespecified follow-up-time analyses suggested that the association was concentrated during early follow-up. However, the magnitude and statistical significance of the association varied across analytical specifications, with attenuation to non-significance in some clinically relevant analyses, including the 2-hospital-day lag model and additional adjustment for vasopressor use. Some models incorporating acute illness-severity factors could not be reliably estimated. These findings indicate substantial sensitivity to exposure timing and confounding control and reinforce the possibility of residual confounding by cardiovascular disease severity, hemodynamic instability, and treatment intensity. Accordingly, the observed association should be interpreted as a hypothesis-generating safety signal rather than evidence of a direct nephrotoxic effect of metoprolol or a basis for changing clinical prescribing. Further studies with higher-resolution exposure, hemodynamic, and renal-function data are needed to determine whether the association is causal.

## Data Availability

The raw data supporting the conclusions of this article will be made available by the authors, without undue reservation.
